# Baihe Dihuang Tang as a therapeutic candidate for insomnia: Targeting gut dysbiosis and neuroendocrine dysfunction

**DOI:** 10.1002/imo2.60

**Published:** 2025-02-03

**Authors:** Liuxi Chu, Qin Lu, Pingping Chen, Zunyong Feng, Ping Wu, Jiamen Shen, Yi Jiang, Yang Yang, Xiran Tan, Xiaomeng Wang, Guoxing Deng, Xi Wang, Xiaokun Li, Zhouguang Wang

**Affiliations:** ^1^ Affiliated Cixi Hospital Wenzhou Medical University Cixi Zhejiang China; ^2^ National Key laboratory of macromolecular drug development and manufacturing, School of Pharmaceutical Science Wenzhou Medical University Wenzhou Zhejiang China; ^3^ School of Basic Medical Sciences Hebei University of Chinese Medicine Shijiazhuang Hebei China; ^4^ Graduate School Hebei University of Chinese Medicine Shijiazhuang Hebei China; ^5^ College of Pharmacy Hebei University of Chinese Medicine Shijiazhuang Hebei China

**Keywords:** Baihe Dihuang Tang, gut microbiota, insomnia, neuroendocrine systems, traditional Chinese medicine

## Abstract

Baihe Dihuang Tang (BDT), is a traditional Chinese medicinal formulation historically utilized to manage various health conditions, including insomnia. This therapeutic use of BDT for treating insomnia is rooted in its potential to regulate the gut microbiota, neuroendocrine, and serotonin systems, which may collectively contribute to its effectiveness. This study aims to explore the anti‐insomnia effects of BDT, and focus on its underlying mechanisms, emphasizing the potential interplay with gut microbiota, neuroendocrine, and serotonin pathways. An insomnia mouse model was induced using p‐chlorophenylalanine (PCPA). Subjects received varying doses of BDT or a saline solution as a control. Behavioral assessment was conducted via the open field test and elevated plus maze test. Hypothalamic monoamine neurotransmitter levels were quantified using ELISA kits. Neurosteroid levels in brain and serum samples were determined through high‐performance liquid chromatography‐tandem mass spectrometry (HPLC‐MS/MS). Gut microbiota composition was evaluated using 16S rRNA amplicon sequencing. PCPA‐induced insomnia led to significant alterations in neurosteroids, monoamine neurotransmitters, and gut microbiota composition. BDT treatment markedly improved behavioral parameters in insomniac mice, evidenced by enhanced motility and reduced sleep latency compared to controls. BDT administration restored neurosteroid and monoamine neurotransmitter dose‐dependently, suggesting potential for neuroendocrine system homeostasis restoration. BDT‐treated mice exhibited significant gut microbiota composition changes, including reduced Acidobacteria, increased Fusobacteria and Firmicutes at the phylum level, and decreased Alistipes at the genus level, compared to the insomnia model group. BDT effectively rectifies gut dysbiosis and mitigates neuroendocrine and serotonin system dysfunctions induced by insomnia, emerging as a promising therapeutic candidate for insomnia management.

## Highlights

- This study demonstrates that Baihe Dihuang Tang (BDT) effectively corrects gut dysbiosis linked to insomnia, suggesting its potential to improve sleep quality through microbiota regulation.
- BDT shows the ability to normalize neurosteroid and monoamine neurotransmitter levels in hypothalamus, indicating its multifaceted effects on neuroendocrine dysregulation in sleep disorders.
- The observed improvements in locomotion and reduced sleep latency in insomniac mice treated with BDT highlight its comprehensive therapeutic potential for addressing both behavioral and biochemical factors of insomnia.

## Graphical abstract

In this study, we utilized a *PCPA*‐induced insomnia mouse model to investigate the therapeutic effects of Baihe Dihuang Tang (BDT) on sleep disorders. Our findings indicate that BDT treatment effectively reshapes the dysregulated gut microbiota, characterized by a decrease in *Acidobacteria* and an increase in *Fusobacteria* and *Firmicutes* at the phylum level. Additionally, BDT treatment restores the abnormal levels of neurotransmitters in various brain regions and corrects the hormonal imbalances observed in peripheral blood. Behavioral assessments demonstrated marked improvements in locomotion and reduced sleep latency among BDT‐treated mice compared to the insomnia model group. These results highlight the potential of BDT as a promising therapeutic agent for insomnia, suggesting that its multifaceted mechanisms may involve the modulation of gut microbiota and restoration of neuroendocrine and serotonin system functions. Overall, our findings provide compelling evidence supporting BDT's role in the management of insomnia, paving the way for further exploration in clinical applications.

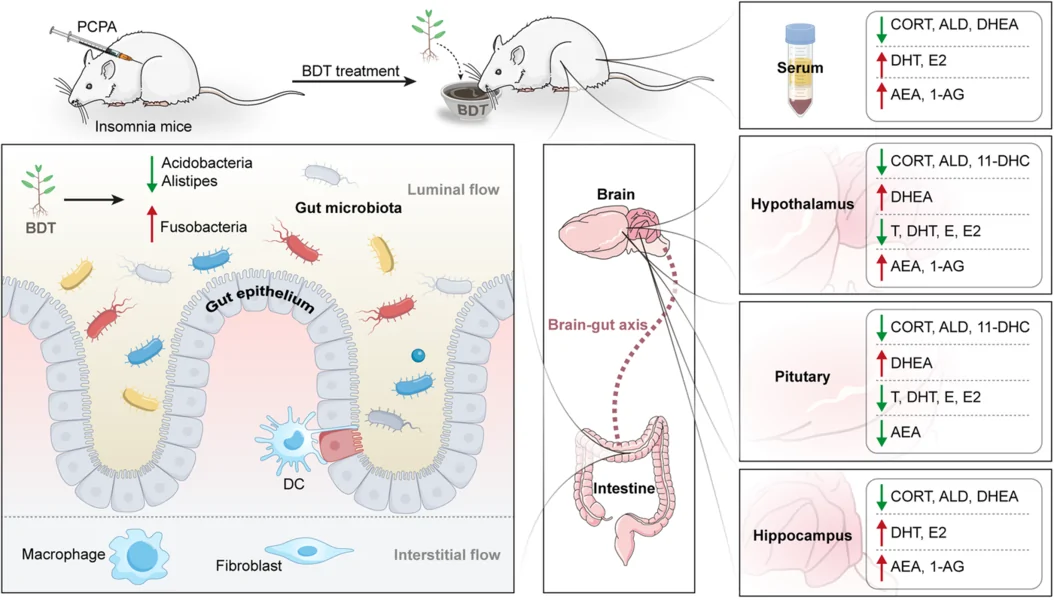

## 1 INTRODUCTION

Insomnia, a prevalent sleep disorder, affects approximately 6%–10% of adults globally [1]. This condition emerges from a complex interplay of environmental, cultural, and genetic factors, significantly impacting both physical and mental health. It is closely associated with an increased prevalence of anxiety, depression, and other mental health disorders [2, 3], as well as elevated risks of obesity, diabetes, stroke, and coronary artery disease [4, 5]. Consequently, elucidating the underlying mechanisms of insomnia is imperative for developing effective treatments to enhance the quality of life for affected individuals.

Emerging research highlights a bidirectional interaction between gut microbiota and the hypothalamus‐pituitary‐adrenal (HPA) axis, a key endocrine system responsive to stress, significantly influencing insomnia [6]. The gut microbiota has been shown to affect sleep quality through mechanisms involving the HPA axis. Our previous study indicated that variations in microbial composition modulate the activity of the HPA axis [7], thereby influencing corticosterone levels and stress responses, which are pivotal in sleep regulation [8]. Conversely, chronic sleep disruption can alter gut microbiota composition, disrupting gastrointestinal function and further unbalancing the HPA axis [9, 10]. Therefore, understanding the complex relationship between gut microbiota and the HPA axis is crucial for developing novel therapeutic strategies for treating insomnia.

Furthermore, insomnia, often associated with heightened stress levels, can induce abnormalities in the HPA axis and may also affect the activities of the hypothalamus‐pituitary‐gonadal (HPG) axis and the endocannabinoid system (ECS). These three endocrine systems, highly sensitive to stress, exhibit intricate interconnections, as highlighted in our previous research [7, 11, 12, 13, 14]. We assessed HPA axis activity through concentrations of corticosterone (CORT) and its metabolite, 11‐dehydrocorticosterone (11‐DHC), and aldosterone (ALD) [13]. The HPG axis was evaluated using levels of testosterone (T), androstenedione (A4), and dihydrotestosterone (DHT), and estrogens (estradiol (E2), estrone (E1)) and progesterone (P) [15, 16]. ECS activity was determined by measuring endocannabinoids such as anandamide (AEA) and 1‐arachidonoylglycerol (1‐AG) [11, 12]. Insomnia impacts the HPG axis, which is crucial for reproductive health and linked with stress‐related disorders [6], and the ECS, a key homeostasis regulator that becomes dysregulated during prolonged sleep disturbances [8], is multifaceted. However, research exploring the simultaneous effects of insomnia on these three endocrine systems—HPA, HPG axes, and ECS—remains limited. This knowledge gap underscores the need for comprehensive research to gain innovative insights into this complex interaction and highlights the necessity of a holistic approach to developing effective insomnia treatment strategies. Additionally, the regulation of neurotransmitters such as serotonin, norepinephrine, and glutamic acid has also been closely linked with the pathogenesis of insomnia [17].

Current clinical treatments for insomnia predominantly rely on pharmacological agents, which often result in numerous adverse effects. Therefore, there is a pressing need to explore natural substances with anti‐insomnia properties and minimal side effects. The use of complementary and alternative medicine, particularly traditional Chinese medicine (TCM), in treating insomnia has gained increasing attention [18, 19, 20]. Baihe Dihuang Tang (BDT), a TCM herbal decoction, has been extensively used in China since 200 AD for the treatment of “Baihe disease,” which presents symptoms similar to anxiety disorders, including anxiety, fear, muscle tension, and apprehension [21]. The formula, documented in the “Synopis of Prescriptions of the Golden Chamber” by Zhongjing Zhang and comprises two herbs: *Lilium lancifolium* Thunb and *Rehmannia glutinosa* (Gaertn.) DC. (http://www.worldfloraonline.org). Although recent advancements in preclinical and clinical practice have shown that BDT is commonly used to manage insomnia [22, 23, 24], the precise mechanisms of BDT's efficacy in treating insomnia are not fully understood.

Extensive research has demonstrated that TCM significantly contributes to the proliferation of probiotics and suppression of pathogenic bacteria, thereby maintaining gut microbiota homeostasis and addressing neurotransmitter imbalances [25, 26, 27]. Given the involvement of the gut microbiota and endocrine systems in the pathogenesis of insomnia, BDT may positively influence sleep quality through interactions with the HPA, HPG axes, ECS, and serotonin system, as well as gut microbiota. Further research is required to comprehensively understand the mechanisms by which BDT and other traditional medicines may improve sleep quality and address insomnia.

To explore the potential of BDT in rectifying insomnia‐induced neuroendocrine dysregulation, we hypothesized a model centered on the modulation of key hormonal axes. In this study, we employed a PCPA‐induced insomnia mouse model to examine the anti‐insomnia mechanism of BDT. This model was designed to elucidate the potential relationship between gut microbiota imbalances, neuroendocrine system dysfunction, and behavioral abnormalities in rodents. We conducted various tests, including the open field test (OFT), elevated plus maze test (EPMT), tail suspension test, pentobarbital‐induced falling asleep rate, latency of sleeping time, and duration of sleeping time experiments, to evaluate the behavior of the mice. Additionally, the concentrations of the previously mentioned 12 neurosteroids were analyzed using HPLC‐MS/MS. Results indicated that BDT treatment ameliorated the behavior of PCPA‐induced mice, including increases in body weight and improvements in depressive and anxious behaviors. Notably, the gut microbiota diversity significantly improved, characterized by an increased abundance of *Lachnospiraceae* and a decreased in *Bacteroideceae*. These findings offer a novel perspective for TCM in treating insomnia, suggesting that BDT may have a promising role in promoting sleep.

## 2 RESULTS

### Identification of the chemical composition of BDT extracts

**FIGURE 1:**
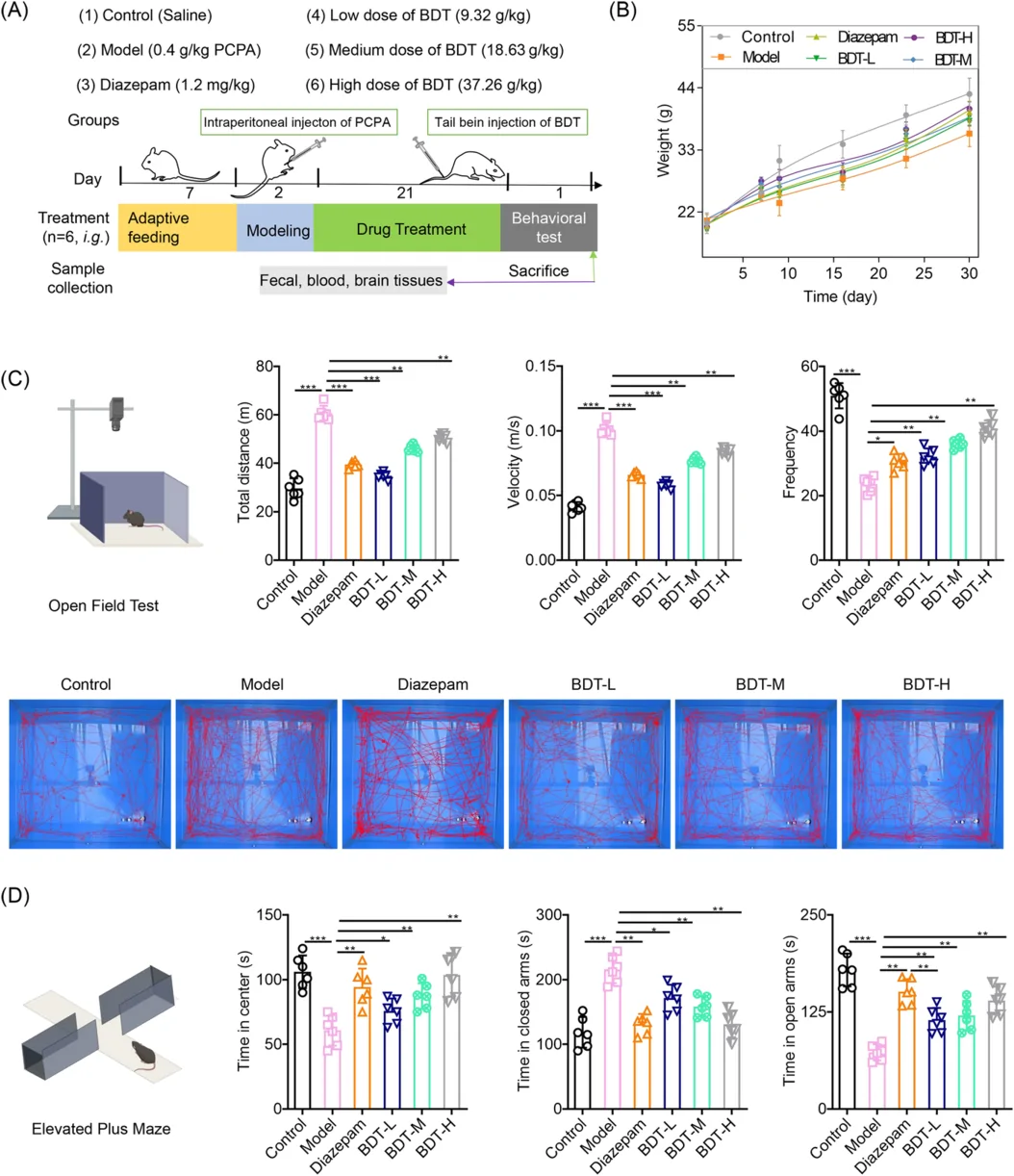
Effects of BDT on various behaviors in the *PCPA*‐induced insomnia model for anxiety. A schematic illustration of the experimental protocol is shown in (A). The graphs demonstrate the influence of BDT on body weight (B). Performance in the open field test (C). Elevated plus maze test (D). Data are presented as mean ± SEM (*n* = 6). Statistical significance is denoted by *p < 0.05, **p < 0.01, ***p < 0.001.

Using UPLC‐QTOF‐MS analysis in both positive and negative modes, we acquired the total ion flow diagram for BDT. Data collection and processing were performed using Compound Discoverer 3.1.0. software. Multiple confidence criteria were employed, including mass accuracy, retention time, area, and notably, library matching based on the mzVault database. This method aimed to compressively screen the target compounds without standard products. Ingredients within BDT were successfully identified (Figure 1 and Tables S1, and S2).

### BDT treatment improved the anxiety behaviors in mice

A schematic diagram illustrating the experimental procedure is depicted in Figure 1A. After a week of acclimatization, mice were treated with PCPA for two consecutive days, exhibiting increasing anxiety‐like behaviors and reduced body weight gain compared to the control group. BDT treatment indicated a trend towards recovery in body weight (Figure 1B). In the OFT and EPMT, insomnia model mice showed decreased time spent in central and non‐edge areas and increased time in edge areas (*p* < 0.05), indicative of heightened anxiety. BDT treatment alleviated these anxiety symptoms in a dose‐dependent manner (Figure 1C). EPMT results further demonstrated BDT's effectiveness in reducing anxiety‐like behaviors, with treated mice spending more time in the open arms and less in the closed arms than the insomnia model mice (*p* < 0.01) (Figure 1D).

### The effect of BDT on hypothalamic neurotransmitters

**FIGURE 2:**
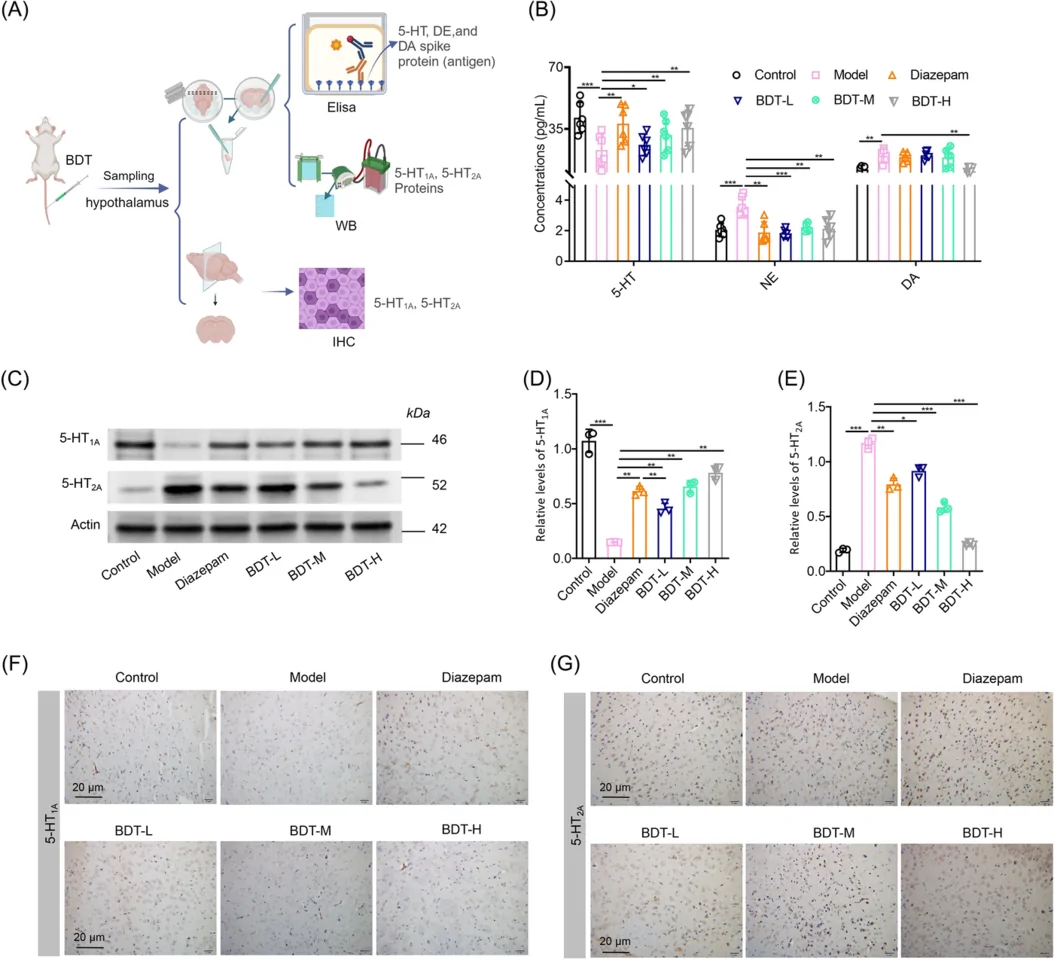
Effect of BDT on monoamine neurotransmitters and the expressions of 5‐HT_1A_ and 5‐HT_2A_ in hypothalamus of insomnia mice. The experimental protocol is shown in the figure labeled (A). The level of serotonin (5‐HT), norepinephrine (NE), and dopamine (DA) in hypothalamus was measured by Elisa kits (B). The protein expressions of 5‐hydroxytryptamine receptor 1A (5‐HT_1A_) and 5‐hydroxytryptamine receptor 2 (5‐HT_2A_) (C). Quantification of protein levels of 5‐HT_1A_ (D) and 5‐HT_2A_ (E) in hypothalamic tissue. The expressions of 5‐HT_1A_ (F) and 5‐HT_2A_ (G) in hypothalamic tissue were stained with immunohistochemical of 200×. Data are presented as mean ± SEM (*n* = 3). Statistical significance is denoted by *p < 0.05, **p < 0.01, ***p < 0.001.

Analysis of monoamine neurotransmitters in the hypothalamus revealed significant variations (Figure 2A–C). Compared to the control group, insomnia model mice had decreased levels of serotonin (5‐HT) and increased of dopamine (DA), and norepinephrine (NE) (*p* < 0.01). BDT and diazepam treatments significantly increased 5‐HT levels and decreased NE production in a dose‐dependent manner, with BDT‐H notably reducing DA levels (*p* < 0.01). 5‐hydroxytryptamine receptor 1A (5‐HT_1A_) and 5‐hydroxytryptamine receptor 2A (5‐HT_2A_) are critical for the regulation of sleep. We also investigated the expression of the two proteins in the hypothalamus of insomnia mice by immunohistochemistry staining and WB (Figure 2D–G). Compared to the control group, 5‐HT_1A_ protein expression decreased while 5‐HT_2A_ protein increased in insomnia mice (*p* < 0.01). BDT and diazepam treatments upregulated 5‐HT_1A_ and downregulated 5‐HT_2A_ protein expression (*p* < 0.01), suggesting BDT's role in enhancing sleep by modulating neurotransmitter secretion.

### BDT‐induced reversal of neurosteroids imbalances in the hypothalamus and pituitary of insomnia mice

**FIGURE 3:**
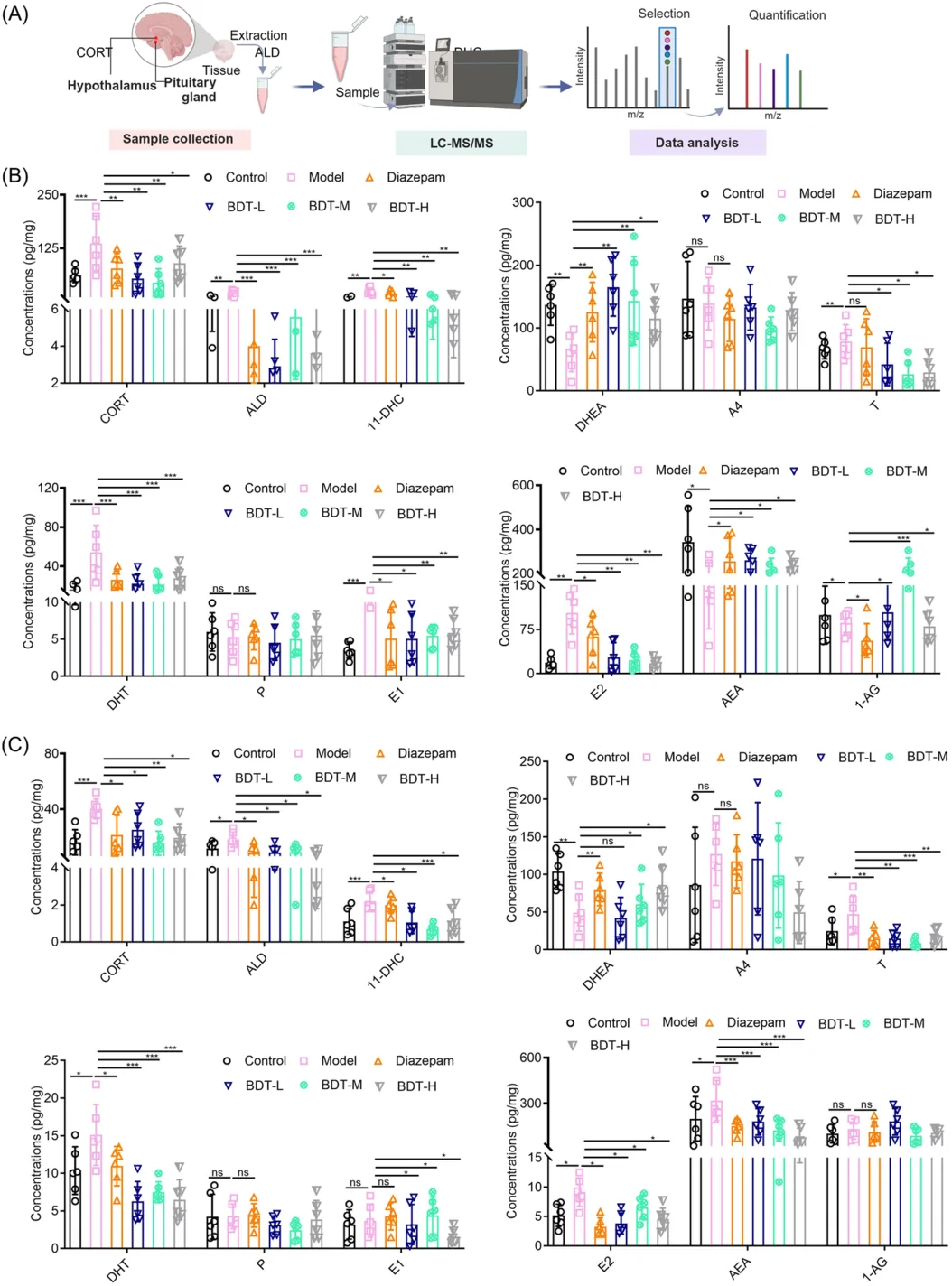
Levels of neurosteroids in different brains of Mice. A schematic diagram of the experimental protocol is provided in (A). The levels of 12 neurosteroids in the hypothalamus (B) and pituitary (C). *Note*: Corticosterone (CORT), aldosterone (ALD), 11‐dehydrocorticosterone (11‐DHC), dehydroepiandrosterone (DHEA), androstenedione (A4), testosterone (T), dihydrotestosterone (DHT), progesterone (P), estrone (E1), estradiol (E2), *N*‐arachidonoyl ethanolamide (AEA), and 1‐arachydonoyl glycerol (1‐AG). Data are expressed as mean ± SEM (*n* = 6). *p < 0.05, **p < 0.01, ***p < 0.001 were considered to indicate significant difference.

To investigate whether BDT can reverse the levels of neurosteroids in the hypothalamus and pituitary induced by insomnia, we measured the levels of 12 neurosteroids in these two brain regions (Figure 3A).

In the hypothalamus of the insomniac mice, levels of corticosterone (CORT) and its metabolites, ALD, and 11‐dehydrocorticosterone (11‐DHC) were significantly elevated compared to the control group (*p* < 0.01). Posttreatment, BDT, and diazepam markedly reduced levels of CORT, ALD, and 11‐DHC (*p* < 0.05), suggesting an over‐activation of the HPA axis in insomniac mice and the ability of BDT to rebalance this axis. The insomniac mice also exhibited significantly reduced levels of dehydroepiandrosterone (DHEA) and increased levels of testosterone (T), dihydrotestosterone (DHT), estrone (E1), and estradiol (E2) compared to controls (*p* < 0.05). Treatment with BDT and diazepam, in a dose‐dependent manner, effectively increased DHEA secretion and decreased T, DHT, E1, and E2 levels (*p* < 0.05), indicating an over‐activation of the HPG axis in insomniac mice and the efficacy of BDT in correcting this imbalance. Additionally, in the insomniac model, neurosteroids related to the ECS, specifically N‐arachidonoyl ethanolamide (AEA) and 1‐arachidonoyl glycerol (1‐AG), displayed marked dysregulation, as evidenced by their significantly reduced levels (*p* < 0.05). BDT administration led to a dose‐dependent improvement in the balance of these endocannabinoids (*p* < 0.05) (Figure 3B), indicating dysfunction of the ECS in the insomniac mice and the potential of BDT in ameliorating this imbalance.

In the pituitary, a similar pattern of neurosteroid changes to that observed in the hypothalamus was noted, with an exception for 1‐AG levels. In the hypothalamus of insomniac mice, 1‐AG levels were significantly lower compared to the control group, a discrepancy that was rectified following BDT treatment (*p* < 0.05). However, in the pituitary region, there was no significant difference in 1‐AG levels between the insomniac and control group (Figure 3C).

The results outlined above demonstrate a clear therapeutic effect of BDT in correcting neuroendocrine disturbances associated with insomnia. By normalizing neurosteroid levels across key endocrine pathways, BDT emerges as a potential therapeutic agent in the management of insomnia, underscoring the significance of neuroendocrine homeostasis in sleep disorder pathophysiology. These findings support further targeted research to unravel the specific mechanisms by which BDT modulates neuroendocrine function and to explore its clinical application in insomnia treatment.

### Normalization of hippocampal and serum hormonal imbalances by BDT treatment

**FIGURE 4:**
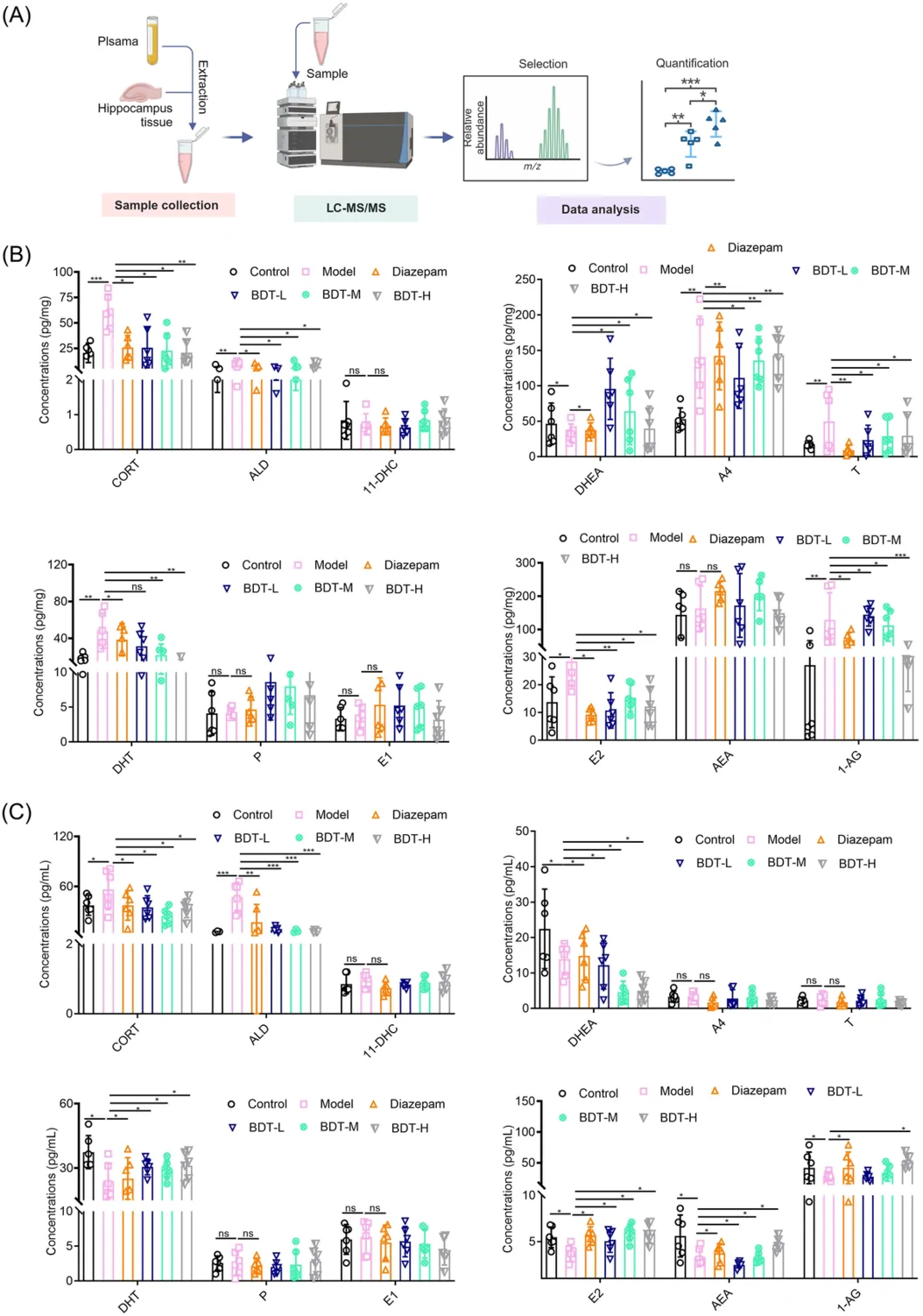
Levels of neurosteroids in the central and peripheral of Mice. The schematic in (A) outlines the experimental protocol. Contents of neurosteroids in the hippocampus (B) and plasma (C). Data are expressed as mean ± SEM (*n* = 6). *p < 0.05, **p < 0.01, ***p < 0.001 were considered to indicate significant difference.

To thoroughly understand neuroendocrine dysfunctions in insomnia, focusing on crucial regulatory systems like the HPA and HPG axes, and the ECS, we measured 12 hormonal levels in the hippocampus and serum (Figure 4A). In the hippocampus, insomniac mice showed significantly elevated levels of CORT and ALD compared to the control group (*p* < 0.05). In contrast, these mice had markedly lower DHEA levels but significantly higher levels of androstenedione (A4), T, DHT, and E2 than controls (*p* < 0.05). Additionally, the insomnia group exhibited substantially higher levels of 1‐arachidonoylglycerol (1‐AG) compared to controls (*p* < 0.01). Post‐BDT treatment, we observed a notable decrease in CORT and ALD levels in the insomnia group, with the high‐dose BDT treatment (BDT‐H) showing the most significant reduction (*p* < 0.05), paralleling trends in the hypothalamus and pituitary. The levels of DHEA, A4, T, DHT, and E2 approached normalization, particularly with medium and high doses of BDT. Furthermore, Posttreatment levels of 1‐AG were significantly diminished (Figure 4B). Similarly, insomniac mice demonstrated significantly higher serum levels of CORT and ALD compared to controls (*p* < 0.05) while exhibiting notably lower serum levels of DHEA, DHT, and E2 (*p* < 0.05). Notably, serum levels of AEA and 1‐AG were significantly reduced in insomniac mice compared to controls (*p* < 0.05). Posttreatment with all three BDT doses and diazepam markedly reduced serum levels of CORT and ALD (*p* < 0.05) and significantly elevated serum levels of DHEA, DHT, E2, AEA, and 1‐AG (*p* < 0.05) (Figure 4B).

These findings suggest that BDT treatment broadly modulates neurosteroids in both central and peripheral tissues, indicating a potential mechanism for its therapeutic efficacy in treating insomnia. This study underscores the comprehensive nature of neuroendocrine modulation by BDT and emphasizes the importance of considering both central and peripheral neurosteroid profiles in understanding the pathophysiology of insomnia.

### Induction of gut dysbiosis by PCPA‐triggered insomnia

**FIGURE 5:**
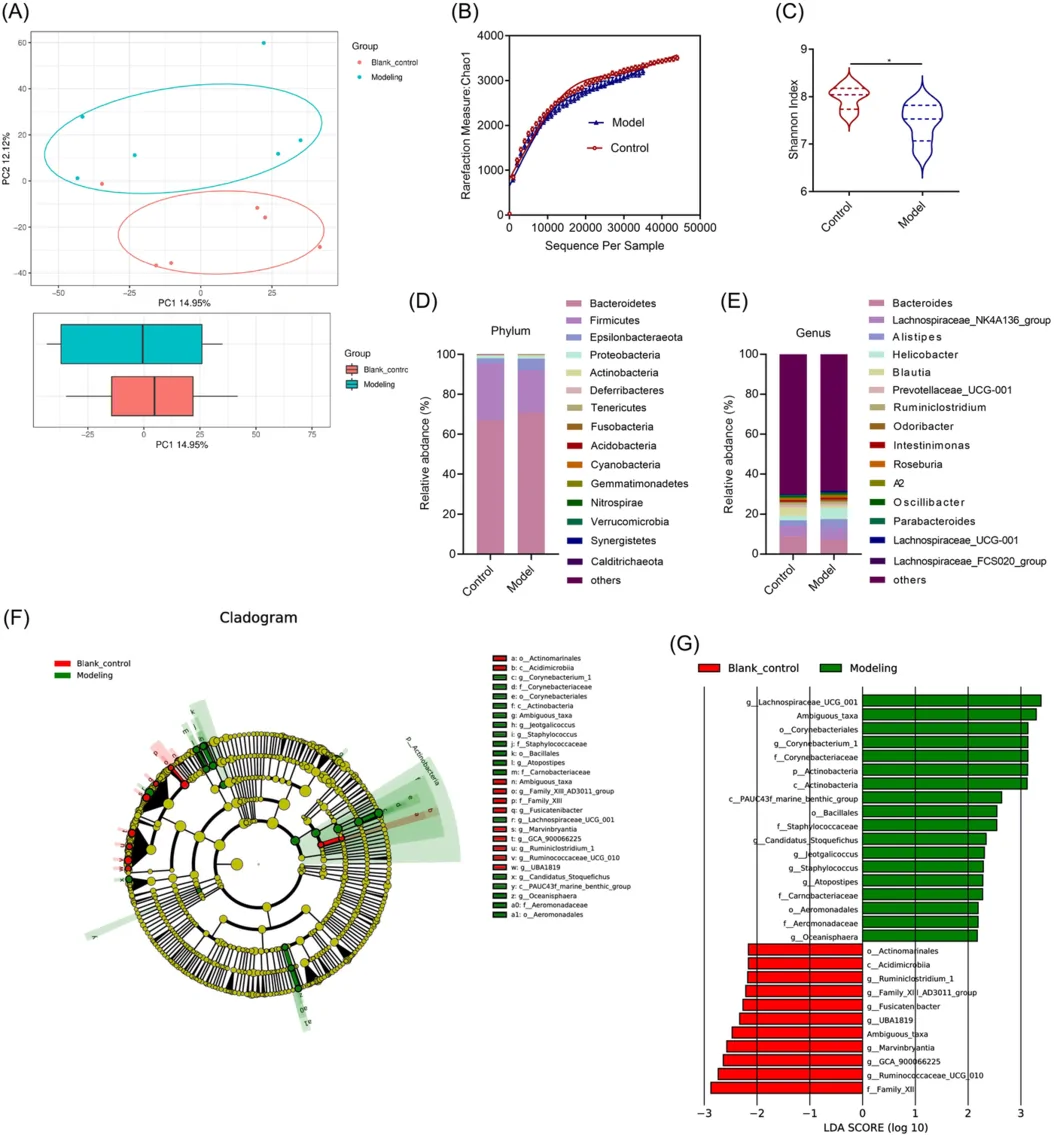
Gut dysbiosis caused by PCPA‐triggered insomnia. (A) PC analysis between control and model groups. (B) Chao1. (C) Shannon index. (D, E) The relative abundance of gut microbiota in phylum and genus levels. (F) Cladogram indicating the phylogenetic distribution of microbiota correlated with the control and insomnia groups. (G) Linear discriminant analysis (LDA) integrated with effect size (LEfSe) between the control and insomnia groups.

To evaluate the impact of para‐chlorophenylalanine (PCPA)‐induced insomnia on the gut microbiota in mice, fecal samples were analyzed using 16S rRNA gene sequencing. This analysis revealed significant alterations in the composition of the microbial community and a notable shift in the alpha diversity of gut microbiota (Figure 5A,C) following two consecutive days of PCPA‐induced insomnia, administered via intraperitoneal injection. The saturation plateau of the rarefaction curves indicated sufficient sequencing depth to capture the full extent of bacterial diversity (Figure 5B). At the phylum level, Bacteroidetes and Firmicutes predominated in both the control and insomnia‐affected mice (Figure 5D), with Bacteroidetes representing 66.94% and 70.73%, and Firmicutes accounting for 28.56% and 21.41% of the sequences in the control and insomnia group, respectively (Table S3). Genus‐level analysis classified sequences into 15 genera (Figure 5E), with Bacteroidetes and Lachnospiraceae as the dominant genera. Bacteroidetes comprised 8.94% and 7.13%, and Lachnospiraceae 5.22% and 5.88% of sequences in the control and insomnia groups, respectively (Table S4). To discern specific bacteria associated with insomnia, LEfSe analysis was employed, resulting in a cladogram identifying 29 discriminatory gut microbiotas at the genus level as key discriminants (Figure 5F). In the insomnia group, Lachnospiraceae, Ambiguous, Corynebacterium, Corynebacteriaceae, and Actinobacteria were significantly overrepresented (all LDA scores (log_10_) > 3), while Ambiguous, Marvinbryantia, and Ruminococcaceae were most abundant in the control group (LDA scores (log_10_ > 2.6), as depicted in Figure 5G. This analysis underscores the profound dysbiosis in gut microbiota induced by insomnia.

### BDT treatment efficacy in reversing gut dysbiosis induced by PCPA‐triggered insomnia

**FIGURE 6:**
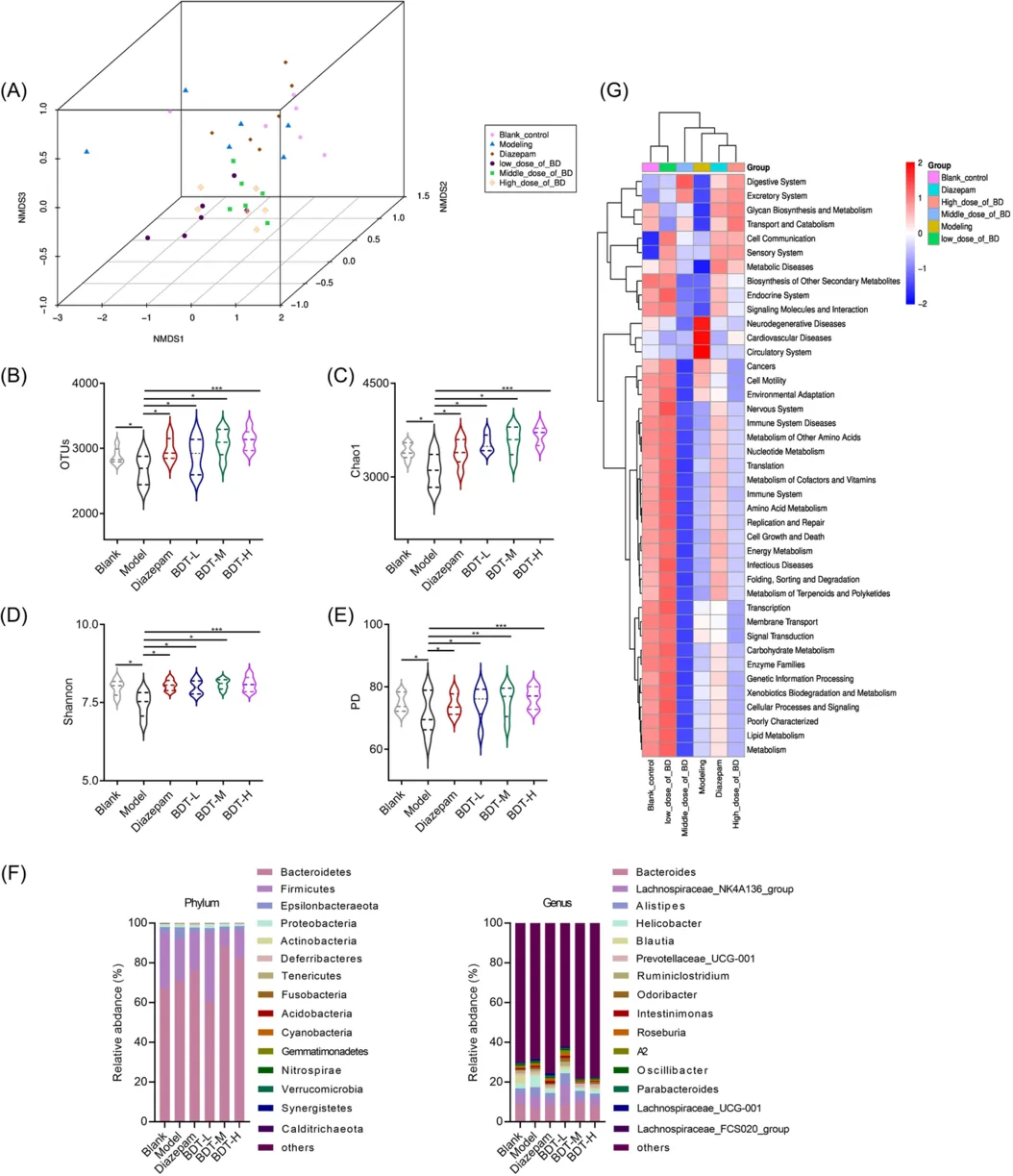
BDT treatment shape gut dysbiosis. (A) PCoA analysis among six groups. (B) OTUs. (C) Chao1 index. (D) Shannon index. (E) PD index. (F) Relative abundance of bacterial in the genus and phylum levels. (G) Signaling pathway among the six groups. OTUs, operational taxonomic units.

**FIGURE 7:**
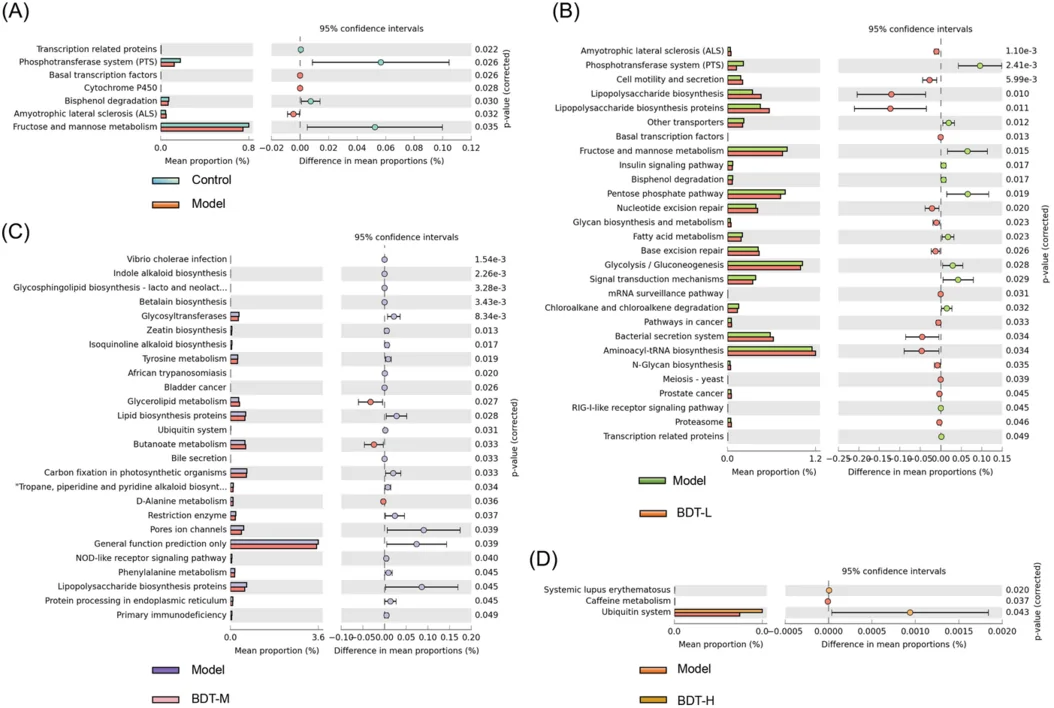
Comparative analysis of different doses of Baihe Dihuang Tang (BDT) groups with model group in signal pathways. (A) Blank control group and model group; (B) low‐dose BDT group and mode group; (C) medium‐dose BDT and model group; (D) high‐dose BDT and model group.

16S rRNA gene sequencing assessed the effect of BDT on gut microbial composition in insomniac mice. BDT altered the gut microbial composition across various taxonomic levels, suggesting its role in combating insomnia. A total of 73,790 high‐quality reads were obtained, clustered into 938 OTUs at 97% sequence similarity. NMDS analyses revealed distinct microbial community patterns among the group, with BDT and diazepam treatments aligning closer to the control group (Figure 6A). Alpha diversity indices reflected enhanced microbial diversity following BDT treatment (Figure 6B–E). Significant changes in gut microbiota composition were observed at all taxonomic levels, with Bacteroidetes and Firmicutes being the main microflora (Figure 6F). LEfSe and LDA analyses further confirmed significant changes in the gut microbiome composition (Figure 6G, Figure S3A–S3B). Notably, BDT treatment influenced Acidobacteria and Fusobacteria levels in a dose‐dependent manner (Figure S2A–S2B). KEGG pathway enrichment analysis identified significant differences in cell communication, sensory system, and metabolic diseases pathways among the group (Figure 7).

### Correlation analysis between differential neurosteroids in different brain regions and serum and gut microbiota

**FIGURE 8:**
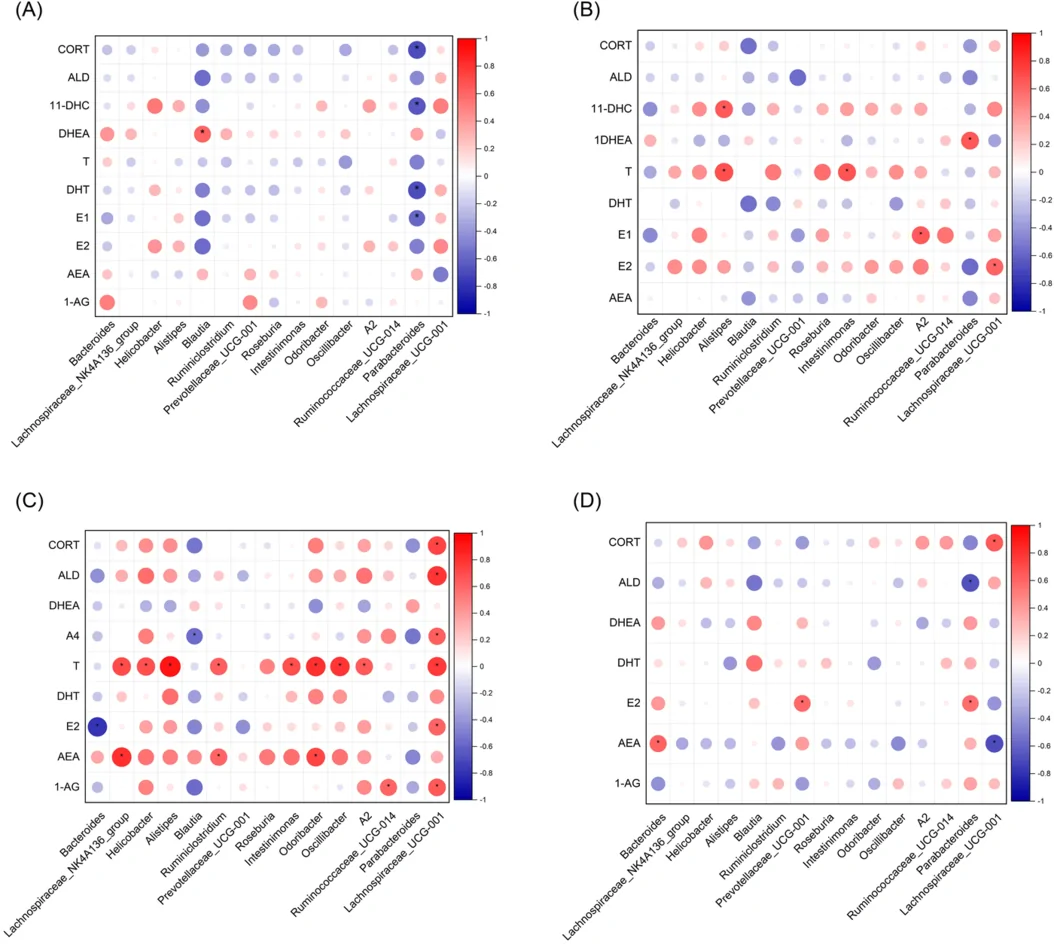
Correlation analysis between the relative abundance of top 15 genus in the gut microbiota and differential neurosteroids in the different brain regions and blood. Correlation between the abundance of intestinal flora at the genus level and neurosteroids in the hypothalamus (A), pituitary (B), hippocampus (C), and serum (D). *Note*: *p < 0.05; **p < 0.01; ***p < 0.001.

*Spearman* correlation analysis was performed to understand the relationship between differential neurosteroids in the hypothalamus, pituitary, hippocampus, and serum with gut microbiota at the genus level (Figure 8). Positive correlations were observed between Blautia and DHEA, and negative correlations between Parabacteroides with various neurosteroids in the hypothalamus (Figure 8A). In the pituitary, positive correlations were found between certain neurosteroids and microbial genera like Alistipes and Intestinimonas (Figure 8B). The hippocampus showed the most significant correlation with gut microbes, particularly Lachnospiraceae_UCG_001, which was positively correlated with several neurosteroids (Figure 8C). Bacteroides exhibited a positive correlation with AEA, while Lachnospiraceae_UCG_001 showed a negative correlation (Figure 8D). These findings highlight a close relationship between gut microbiota and neuroendocrine systems in the regulation of insomnia.

## 3 DISCUSSIONS

In this study, we extensively investigated the anti‐insomnia effects of BDT on p‐chlorophenylalanine (PCPA)‐induced insomnia‐like behaviors, focusing on the mediation of these effects through various neuroendocrine systems, including the HPA axis, HPG axis, and the ECS, the serotonin system, and the gut microbiota. Our findings reveal that PCPA‐induced insomnia disrupts neurosteroids, monoamine neurotransmitters, and gut microbiota composition. Importantly, BDT treatment showed a dose‐dependent normalization in neurosteroid and monoamine neurotransmitter levels, suggesting its efficacy in restoring neuroendocrine systems homeostasis. Additionally, BDT‐treated mice exhibited substantial changes in gut microbiota composition, particularly a decrease in *Acidobacteria* and an increase in *Fusobacteria* and *Firmicutes* at the phylum level, and a reduction in *Alistipes* at the genus level, compared to mice in the insomnia group.

The role of gut microbiota in TCM, as emphasized by Wu et al. [28], is evident in our study. Insomnia mice exhibited altered behavior in the open field and EPMTs, consistent with previous reports on insomnia behavior [29]. However, post‐BDT administration, there was a significant enhancement in autonomic ability, indicating the potential of BDT in alleviating insomnia‐related behavioral phenotypes. This aligns with studies suggesting an association between insomnia and reduced gut microbiota richness and diversity [30, 31]. Our study's alpha diversity analysis further confirms that BDT considerably increases gut microbiota abundance and diversity. Beta diversity analysis revealed that BDT induces changes in gut microbiota structure, showing a restoration towards the normal microbiota composition.

In exploring the microbiomes of fecal samples, we noted remarkable taxonomical differences in the key phyla—*Firmicutes, Bacteroidetes*, and *Actinobacteria*, with *Firmicutes* and *Bacteroidetes* being predominant. The study showed an increased presence of *Acidobacteria* in insomniac mice, which decreased with BDT treatment. Conversely, *Fusobacteria*, associated with certain health conditions [32], were reduced in insomniac mice but increased following BDT treatment. These findings align with previous research [27, 33, 34, 35], highlighting the bidirectional relationship of Firmicutes with health conditions and their role in synthesizing beneficial compounds like butyrate [36]. In addition to this, increasing the level of Fusobacteria can cause infection and disease [32]. The results indicated that BDT increases the *Firmicutes* while reduces *Fusobacteria*, and thus promotes human health, thereby promoting sleep. We also found that, at the genus level, *Alistipes* was significantly more abundant in insomniac mice compared to controls. Following BDT administration, there was a notable dose‐dependent decrease in *Alistipes* populations. *Alistipes*, belonging to phylum *Bacteroidetes*, has been increasingly studied for its potential role in various gastrointestinal disorders. Research has shown that alterations in the abundance of Alistipes are associated with conditions like IBD and irritable bowel syndrome (IBS) [37, 38, 39, 40]. For instance, a study by Schirmer et al. found a significant association between the abundance of *Alistipes* and the severity of IBD [39]. Another study conducted by Su et al. reported a markedly altered abundance of *Alistipes* in patients with IBS compared to healthy controls [40]. More importantly, in our study, the *Spearman* correlation indicated that *Alistipes* was positively correlated with 11‐DHC and T. It is speculated that BDT could modulate the HPA and HPG axes by regulation of gut microbiota. These findings suggest that *Alistipes* may play a role in the pathogenesis or progression of these disorders, potentially through producing short‐chain fatty acids or activating the endocrine systems and thereby impacting the gut's microbial balance and inflammatory processes. However, more research is needed to fully understand its role and mechanisms in gut health and disease.

Furthermore, continuing our comprehensive exploration of BDT's anti‐insomnia properties, we specifically quantified 12 endogenous metabolites to further understand the underlying mechanisms. Our prior research [7, 11, 12, 13, 14] utilized levels of corticosterone (CORT), 11‐dehydrocorticosterone (11‐DHC), and aldosterone (ALD) to assess the activity of the HPA axis. We also assessed the HPG axis using sex hormones, including testosterone (T), androstenedione (A4), dihydrotestosterone (DHT), estrogens (estradiol (E2), estrone (E1)), and progesterone (P) and the ECS via endocannabinoids like anandamide (AEA) and 1‐arachidonoylglycerol (1‐AG). These axes significantly influence sleep patterns and insomnia, with their respective hormonal and neurochemical imbalances playing key roles in sleep disorder development and exacerbation.

Our findings align with the known dynamics of these systems. Chronic stress‐induced activation of the HPA axis leads to elevated levels of corticosterone levels, as observed in our study, along with increased levels of ALD and 11‐DHC, suggesting heightened metabolic activity within the corticosterone pathway and, consequently, HPA axis activation. This increase adversely affects sleep regulations, contributing to insomnia [41, 42]. This finding is consistent with previous research [41, 43, 44], which reported that insomnia is often accompanied by dysregulated HPA axis activity. BDT's ability to normalize these levels highlights its potential in modulating the HPA axis, a crucial factor in the pathology of insomnia and related sleep disorders.

Regarding HPG axis, while primarily associated with reproductive functions, also plays a role in sleep regulation, with its dysregulation potentially contributing to insomnia. For instance, Bracci et al. delved into how testosterone levels affect sleep, highlighting that low testosterone in men is linked to a higher prevalence of sleep disturbances, including insomnia [45]. However, in this study, we found that testosterone levels were indeed elevated in the hypothalamus, pituitary, and hippocampus of insomnia mice compared to the controls, while no difference was observed in serum levels. This inconsistency with previous research might be attributed to the use of different biological substrates and species in our study. Although no difference in testosterone levels was observed in the serum, a variance in the levels of its metabolite dihydrotestosterone was noted. This highlights the limitations of past studies that utilized a single marker to evaluate the activity of the HPG axis. Our approach, employing multiple markers to assess the activity of the HPG axis, demonstrates a more comprehensive and superior method for understanding the intricacies of this endocrine system. Following treatment with BDT, there was a reversal in the levels of these sex hormones, indicating that BDT can correct the imbalance in HPG axis activity caused by insomnia.

Furthermore, our study confirmed the significant role of the ECS in sleep regulation. Consistent with previous research [46], we found reduced levels of AEA and 1‐AG in insomnia mice. This reduction in AEA and 1‐AG is largely due to the antagonistic relationship between the ECS and the HPA axis. Elevated corticosterone levels in these mice indicate a dysregulated HPA axis, leading to suppressed ECS functioning [47]. Such suppression culminates in decreased secretion of endocannabinoids, including AEA and 1‐AG. Our findings suggest that ECS modulation could be a viable approach to managing sleep disorders like insomnia, though further research is required to validate this hypothesis. Besides, our study reveals that behavioral desensitization therapy (BDT) significantly modifies endocannabinoid levels in central (e.g., hypothalamus, hippocampus) and peripheral (e.g., serum) tissues in mice with insomnia. This underscores BDT's potential in correcting hormonal imbalances related to sleep disorders and introduces a potentially novel avenue for therapeutic intervention.

Additionally, monoamine neurotransmitters, particularly dopamine (DA), norepinephrine (NE), and serotonin (5‐HT), play crucial roles in physiological functions [48]. 5‐HT is a key neuromodulator in sleep arousal stage, predominantly promoting wakefulness and inhibiting rapid eye movement sleep (REMS) [49, 50]. 5‐hydroxytryptamine receptor 1A (5‐HT_1A_) and 5‐hydroxytryptamine receptor 2A (5‐HT_2A_), among the 5‐HT receptor subtypes in the hippocampus and hypothalamus regulate sleep by controlling 5‐HT release [51, 52]. Conversely, PCPA acts as a tryptophan hydroxylase inhibitor, impeding 5‐HT synthesis and potentially leading to insomnia [53]. Our study focused on the hypothalamus and revealed that 5‐HT levels and 5‐H_1A_ protein expression were significantly reduced, while 5‐HT_2A_ protein expression was significantly increased in insomnia mice, consistent with prior research [23, 54]. The levels of 5‐HT, NE, and DA maintain a dynamic balance in vivo; elevated brain levels of NE and DA indicate central nervous system excitation. We observed that BDT treatment modulates NE, DA, and 5‐HT levels in the hypothalamus of PCPA‐induced insomnia mice, thus reducing central excitability and enhancing sleep, corroborating earlier findings [23]. These outcomes suggest that the serotonergic system plays a role in the hypnotic effect of BDT.

Our research, offering a holistic perspective, represents a novel contribution to understanding insomnia. Unlike previous studies focusing on singular aspects of complex systems, we examined the interplay between the HPA and HPG axes, the ECS, the serotonin system, and gut microbiota in the context of insomnia. This approach provides a comprehensive understanding of insomnia's pathophysiology, extending beyond traditional focuses on single systems. BDT's modulation of a broad spectrum of neurosteroids and neurotransmitters across these diverse systems offers a systemic therapeutic approach, differing from conventional pharmacological interventions targeting specific pathways. The findings from this study lay the groundwork for future research to further dissect the complex interactions between these systems in sleep regulation and the development of insomnia, with subsequent studies poised to explore BDT's specific components and their mechanisms of action at molecular and cellular levels.

## 4 CONCLUSIONS

This study reveals that BDT significantly ameliorates insomnia by modulating gut microbiota and restoring neuroendocrine and serotonin system balance. BDT administration notably improved behavioral parameters, enhanced neurosteroid and monoamine neurotransmitter levels, and resulted in significant gut microbiota composition changes, including reduced Acidobacteria and increased Fusobacteria and Firmicutes. These findings underscore the potential of BDT as a promising therapeutic candidate for insomnia management, highlighting its effectiveness in rectifying gut dysbiosis and mitigating neuroendocrine dysfunctions. This research not only contributes to the growing body of knowledge in this field but also opens new avenues for future exploration and therapeutic development.

## 5 MATERIALS AND METHODS

### Materials and reagents

Herbal samples of *Lilium lancifolium* Thunb (Lilii Bulbus) and *Rehmannia glutinosa* (Rehmanniae radix) were respectively acquired from Longshan, Hunan Province, and Jiaozuo, Henan Province, China. These were procured through Tongrentang Chinese Pharmaceutical Co. Ltd., Beijing, China. Authenticated voucher specimens for Lilii Bulbus (No. 20211104‐0204LQ) and Rehmanniae radix (No. 20211105‐0205LQ) have been archived at the School of Basic Medical Sciences Herbarium at Hebei University of Chinese Medicine, under the expertize of Prof. Xi Wang.

For the preparation of BDT, we used 30 g of Lily bulb and 20 g of Rehmannia root. Both herbs were coarsely ground with an agate mortar and pestle. This blend was then subjected to a reflux extraction in water at a ratio of 1:8 (w/v) for 30 min at ambient temperature, followed by an additional hour of boiling. Subsequently, the resulting solution was filtered and reduced down to a 200 mL concentrate, which was then split into two equal portions.

Reagents for neurochemical assays were sourced as follows: ELISA kits for serotonin (5‐HT), norepinephrine (NE), and dopamine (DA) were provided by Shanghai Enzyme‐linked Biotechnology Co., Ltd. Antibodies against 5‐hydroxytryptamine receptor 1A (5‐HT_1A_) and 5‐hydroxytryptamine receptor 2A (5‐HT_2A_) (1:500, Cat. BA1391 and DF8900) were purchased from Boster Biological Technology Co. Ltd., and GAPDH antibody (1:1000, Cat. 10494‐1‐AP) was acquired from Hangzhou Dawen Biological Co., Ltd. Chemical and steroid standards such as corticosterone (CORT), aldosterone (ALD), 11‐dehydrocorticosterone (11‐DHC), estradiol (E2), estrone (E1), androstenedione (A4), dihydrotestosterone (DHT), dehydroepiandrosterone (DHEA), testosterone (T), progesterone (P), N‐arachidonoyl ethanolamide (AEA), and 1‐arachydonoyl glycerol (1‐AG), along with their respective internal standards, were sourced from Sigma Aldrich and Cayman Chemical. High‐purity HPLC‐grade ammonium acetate and methanol were obtained from Tedia and Sigma Aldrich, respectively. Ultra‐pure deionized water was supplied by Watsons. Stock solutions for each steroid and lipid mediator were meticulously prepared in methanol to a standard concentration of 100 μg/mL, while a mixed internal standard solution was prepared at 10 μg/mL and stored at −20°C for future assays.

### Animals

A total of 60 male Kun‐Ming strain mice (SPF, 20 ± 2 g, 8 weeks old) were sourced from the Experimental Animal Center of Hebei Medical University (Certificate No. SCXK (jing) 2020‐0017). The mice were housed in controlled conditions with a temperature of 25 ± 2°C, relative humidity of 50 ± 5%, and a 12‐h light/dark cycle (7 AM to 7 PM). They had access to standard laboratory diets and distilled water *ad libitum*. All experimental protocols adhered to internationally accepted principles for laboratory animal use and care as outlined in the European Community guidelines (EEC Directive of 1986; 86/609/EEC) and were approved by the Animal Care Research Advisory Committee of Hebei Medical University (Approval Number: DWLL2021111).

### Establishment of p‐chlorophenyl alanine‐induced insomnia model

After 7 days of adaptive feeding, 60 mice with similar scores, weight, and sugar water consumption were selected using the OFT. Fifty mice were intraperitoneally injected with PCPA suspension (0.4 g·kg‐1) daily for 2 consecutive days at 8:00 AM. The disappearance of circadian rhythm and increased daytime activity indicated successful model replication. These 50 mice were then randomly divided into model groups, diazepam, and BDT with low, medium, and high doses. Ten un‐modeled mice served as the normal control group. Doses for BDT and diazepam were calculated based on human and animal equivalent dose formulas described in the second edition of Experimental Methodology of Pharmacology (1991). Mice in the medium‐dose BDT group received 18.63 g/kg. The dose ratios for the low, medium, and high BDT doses were set as 0.5:1:2, respectively. Diazepam served as the positive control drug. BDT and diazepam were administered intragastrically at 1.2 mg/kg once daily for 21 days. The normal and untreated model group received an equivalent volume of distilled water.

### Behavioral tests

Behavioral assessments were conducted by three trained observers who were blinded to the treatment group. After 21 days of administering either the drug or distilled water via gavage, all animals underwent behavioral testing.

The elevated plus maze (EPM) test, performed 3 days before the end of drug administration [55], involves a maze with two open and two closed arms, each 25 cm long, centering around a 5 × 5 cm square grid. Mice were placed at the center of the open arms, and their activities were recorded for 5 min using a video camera. The analysis focused on the duration and frequency of entering the open arms.

The OFT, conducted as previously described [56], commenced with a 5‐min acclimatization period in the laboratory, followed by a 5‐min behavior monitoring and recording session. The OFT apparatus, measuring 80 × 80 × 20 cm with a grid floor divided into 25 squares, was used to observe the mice. Each mouse was placed in the center for a 3‐min exploration, and the horizontal and vertical activities were scored. The apparatus was sanitized with 75% ethanol after each test.

### Sample collection

Following the behavioral tests, mice were euthanized under anesthesia with a 40 mg/kg intraperitoneal injection of sodium pentobarbital. Blood was collected from the ophthalmic venous plexus and processed for serum separation. Brain tissues (hypothalamus, hippocampus, and pituitary) and fecal samples were also collected, rapidly frozen, and stored at −80°C. Serum and brain tissues were analyzed for neurosteroids, and fecal samples were used to analyze gut microbiota composition.

### Chemical analysis of BDT extracts

UHPLC‐MS/MS analyses were performed using a Vanquish UHPLC system (ThermoFisher) paired with an Orbitrap Q Exactive™ HF mass spectrometer (Thermo Fisher) at Novogene Co., Ltd. BDT extracts were loaded onto a Hypesil Gold column (100 × 2.1 mm, 1.9 μm) and subjected to a 17‐min linear gradient at a flow rate of 0.2 mL/min. For positive polarity mode, eluents A (0.1% FA in water) and B (methanol) were used. For negative polarity mode, eluents A (5 mM ammonium acetate, pH 9.0) and B (methanol) were utilized. The solvent gradient was programmed as follows: 2% B for 1.5 min, a gradient increase from 2% to 100% B over 3 min, maintaining 100% B for 10 min, decreasing from 100% to 2% B from 10.1 to 12 min. The Q Exactive™ HF mass spectrometer operated in both positive and negative polarity modes, with a spray voltage of 3.5 kV, capillary temperature of 320°C, sheath gas flow rate of 35 pi, auxiliary gas flow rate of 10 L/min, S‐lens RF level of 60, and auxiliary gas heater temperature of 350°C. Compound identification was achieved using Compound Discoverer 3.1.0.305 and the mzVault database (http://www.mzcloud.org/).

### Measurement of monoamines neurotransmitters in the hypothalamus

Hypothalamus tissues were processed to measure levels of 5‐HT, DA, and NE using ELISA kits. Tissue homogenates were centrifuged to obtain the supernatant for these measurements.

### Immunohistochemical analysis and western blot of 5‐HT_1A_ and 5‐HT_2A_ in the hypothalamus

The expression of 5‐HT_1A_ and 5‐HT_2A_ receptors in the hypothalamus was evaluated using immunohistochemical staining. Paraffin‐embedded hypothalamic sections were treated with 5‐HT_1A_ and 5‐HT_2A_ antibodies, followed by incubation with a goat anti‐rabbit IgG secondary antibody. Signal visualization was achieved using DAB solution, and nuclei were counterstained with hematoxylin. Positive expression areas appeared yellow under microscopic examination. Protein levels were quantitatively assessed by measuring the integrated optical density in the positively stained regions.

Western blot analysis was used to analyze the expression of 5‐HT_1A_ and 5‐HT_2A_ proteins in the hypothalamus. Hypothalamus was extracted using radioimmunoprecipitation (RIPA) buffer, and protein concentrations were quantified using the BCA protein assay kit. A total of 80 μg of proteins were loaded for gel electrophoresis, followed by their transfer to a PVDF membrane and overnight incubation at 4°C with primary antibodies. After washing, a horseradish peroxidase‐linked secondary antibody IgG (1:5000) was applied to bind the target proteins. Detection was conducted using the chemiluminescence (ECL) Western blot analysis system, with protein expression levels normalized against GAPDH control.

### LC‐MS/MS detection of neurosteroids in brain and serum

In this study, we aim to investigate the concentrations of hormones in various brain regions—including the hypothalamus, pituitary gland, and hippocampus—as well as in peripheral blood under conditions of insomnia. We developed an LC‐MS/MS method to quantify ten hormones and two endocannabinoids in brain and serum samples, inspired by our previous research [57,58]. The method showed robust linearity (*R* ^2^ > 0.99) with limits of quantification ranging from 0.06 to 1.3 pg/mg for brain samples and 0.03–0.6 ng/mL for serum samples. Recovery rates for all compounds ranged from 87.7% to 115.1%in brain samples and 86.1% to 114.6% in serum samples. The method exhibited consistent performance, with intra‐day and inter‐day coefficients of variation below 15%, and demonstrated excellent freeze/thaw and short‐term stability attributes.

### Analysis of 16S rRNA microbial community

For our microbial community analysis, bacterial DNA was isolated from a set of 36 stool samples. We utilized the MagPure Soil DNA LQ Kit (Magen) for DNA extraction, adhering strictly to the instructions provided by the manufacturer. We evaluated the DNA's quality and concentration utilizing a NanoDrop 2000 spectrophotometer (Thermo Fisher Scientific) and checked the DNA integrity through agarose gel electrophoresis.

Amplification of the bacterial 16S rRNA gene's V3‐V4 hyper‐variable regions was conducted using the universal primer pairs 343F (5′‐TACGGRAGGCAGCAG‐3′) and 798R (5′‐AGGGTATCTAATCCT‐3′), which included Illumina sequencing adapters and a barcode unique to each sample. The PCR products were examined by gel electrophoresis, purified using Agencourt AMPure XP beads (Beckman Coulter Co.), and quantified using the Qubit dsDNA assay kit. High‐throughput sequencing was carried out using the Illumina NovaSeq. 6000 platform (Illumina Inc.; OE Biotech Company), generating 250‐base paired‐end reads.

Subsequent data processing involved the use of QIIME2 (version 2019.10) for quality control, demultiplexing, and the DADA2 algorithm for sequence error correction. Chimeric sequences were identified and removed. Non‐chimeric sequences were then clustered into operational taxonomic units (OTUs) at a similarity cutoff of 97%. Taxonomic classification was performed using both the Silva (version 132) and Greengenes (version 13.8) databases. We assessed microbial diversity in the samples using alpha diversity indices, such as Chao1 and Shannon. The phylogenetic relationship and community structure were explored using unweighted Unifrac PCoA and phylogenetic tree construction, with all sequencing and analytical services provided by OE Biotech Co., Ltd.

### Statistical analysis

Data are presented as the mean ± SEM. Statistical analyses were performed using GraphPad Prism Software 7 (GraphPad Software). Comparisons between two groups were made using an unpaired two‐tailed Student's *t*‐test. Comparisons among more than two groups were made using a one‐way ANOVA followed by Dunnett's test. Pearson correlation analysis assessed the relationship between neurosteroid concentrations and gut microbiota diversity. *p* values < 0.05 were considered to indicate significance.

## AUTHOR CONTRIBUTIONS

**Liuxi Chu**: Conceptualization; funding acquisition; writing—original draft; project administration. **Qin Lu**: Software; conceptualization; supervision; writing—original draft. **Pingping Chen**: Software; investigation; writing—original draft. **Zunyong Feng**: Conceptualization; investigation; software; data curation. **Ping Wu**: Validation; methodology. **Jiamen Shen**: Investigation; validation; data curation. **Yi Jiang**: Investigation; formal analysis. **Yang Yang**: Investigation; formal analysis. **Xiran Tan**: Investigation; data curation. **Xiaomeng Wang**: Formal analysis; investigation. **Guoxing Deng**: Writing—review and editing; investigation; conceptualization. **Xi Wang**: Project administration; formal analysis; writing—review and editing. **Xiaokun Li**: Writing—review and editing. **Zhouguang Wang**: Writing—review and editing; project administration.

## CONFLICT OF INTEREST STATEMENT

The authors declare no conflicts of interest.

## 1 ETHICS STATEMENT

The ethics application (No. DWLL2021111) was approved by the Research Ethics Committee of the Hebei Medical University.

## Supporting information

The online version contains supplementary figures and tables available.

**Figure S1:** Total ion chromatograms of Baihe Dihuang Tang (BDT) in both positive and negative modes.
**Figure S2:** Comparison of the differences in the relative abundance of gut microbiota between group at the phylum (A) and genus (B) levels.
**Figure S3:** Phylogenetic distribution and abundance differences of microbiota across six groups.

**Table S1:** The active ingredients of BDT based on high‐performance liquid chromatography‐tandem mass spectrometry (HPLC‐MS/MS) in positive modes.
**Table S2:** The active ingredients of BDT based on HPLC‐MS/MS in negative modes.
**Table S3:** The relative abundance of the gut microbiota in the phylum levels.
**Table S4:** The relative abundance of the gut microbiota in the genus levels.

## Data Availability

The data that support the findings of this study are openly available in BDT treatment efficacy in reversing gut dysbiosis induced by at https://www.ncbi.nlm.nih.gov/bioproject/PRJNA1187478, reference number PRJNAPRJNA1187478. The data that support the findings of this study are available in NCBI of PRJNAPRJNA1187478 (https://www.ncbi.nlm.nih.gov/bioproject/PRJNA1187478). Supplementary materials (figures, tables, graphical abstract, slides, videos, Chinese translated version, and update materials) may be found in the online DOI or iMeta Science http://www.imeta.science/imetaomics/.
