# Supplementary material for: Baihe Dihuang Tang as a therapeutic candidate for insomnia: Targeting gut dysbiosis and neuroendocrine dysfunction
Source: IMetaOmics. 2025 Feb 3;2(1):e60. doi: 10.1002/imo2.60 (PMC12806353; doi:10.1002/imo2.60)
Supplement: Supplementary file 1 — Figure S1: Total ion chromatograms of Baihe Dihuang Tang (BDT) in both positive and negative modes. Figure S2: Comparison of the differences in the relative abundance of gut microbiota between group at the phylum (A) and genus (B) levels. Figure S3: Phylogenetic distribution and abundance differences of microbiota across six groups. [file IMO2-2-e60-s002.docx]

**Supporting information to**

**Baihe Dihuang Tang as a Therapeutic Candidate for Insomnia: Targeting Gut Dysbiosis and Neuroendocrine Dysfunction**

**Running title*: Baihe Dihuang Tang for Insomnia: Modulating Gut and Neuroendocrine Function***

Liuxi Chu ^1, 2#^, Qin Lu ^3, 4#^, Pingping Chen ^3#^, Zunyong Feng ^2^, Ping Wu ^2^, Jiamen Shen ^2^, Yi Jiang ^2^, Yang Yang ^3^, Xiran Tan ^3^, Xiaomeng Wang ^3^, Guoxing Deng ^3*^, Xi Wang ^5*^, Xiaokun Li ^2*^, Zhouguang Wang ^2 *^

^1^Affiliated Cixi Hospital, Wenzhou Medical University, Cixi 31539, Zhejiang, China.

^2^National Key laboratory of macromolecular drug development and manufacturing, School of Pharmaceutical Science, Wenzhou Medical University, Wenzhou 325035, Zhejiang, China.

^3^School of Basic Medical Sciences, Hebei University of Chinese Medicine, Shijiazhuang 050200, Hebei, China.

^4^Graduate School, Hebei University of Chinese Medicine, Shijiazhuang 050091, Hebei, China.

^5^College of Pharmacy, Hebei University of Chinese Medicine, Shijiazhuang 050200, Hebei, China.

^#^ These authors contributed equally: Liuxi Chu, Qin Lu, Pingping Chen

^*^Correspondence: [wangzhouguang@wmu.edu.cn](mailto:wangzhouguang@wmu.edu.cn)(Zhouguang Wang), [xiaokunli@wmu.edu.cn](mailto:xiaokunli@wmu.edu.cn) (Xiaokun Li). [435554030@qq.com](mailto:435554030@qq.com) (Xi Wang), [dengguoxing@hebcm.edu.cn](mailto:dengguoxing@hebcm.edu.cn) (Guoxing Deng).


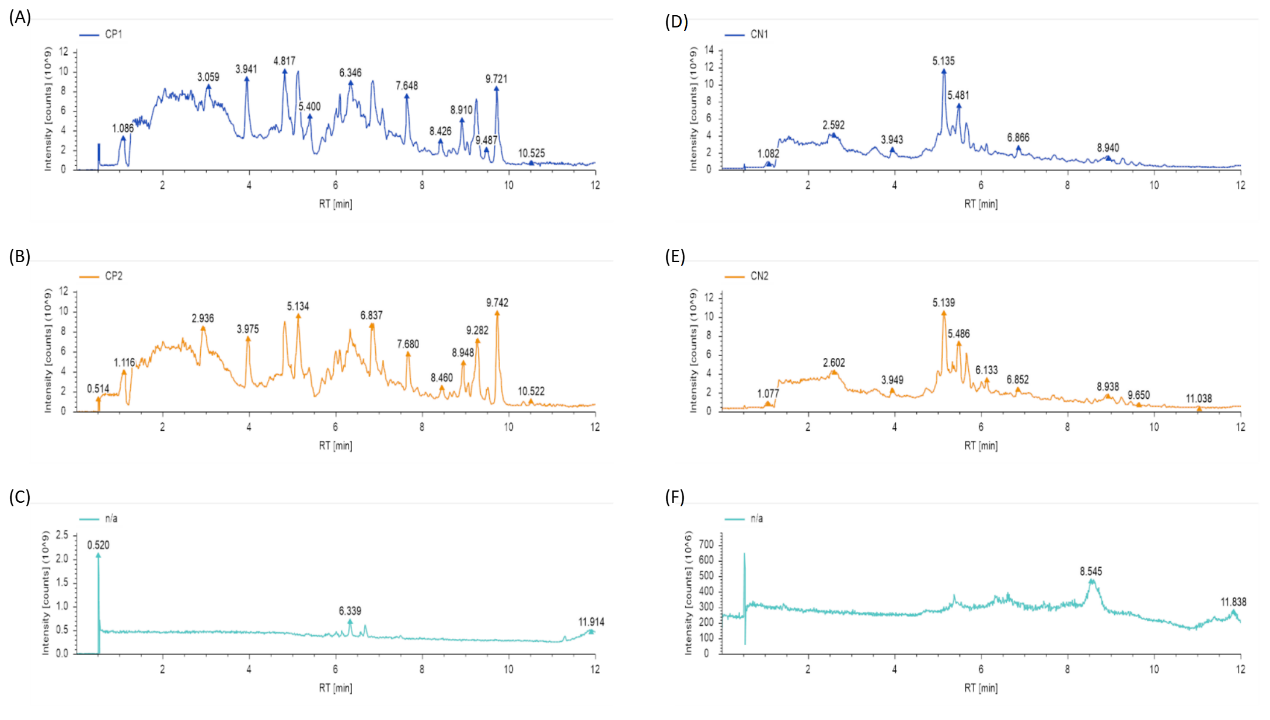


**Figure. S1. Total ion chromatograms of BDT in both positive and negative ion modes.** (A-B) Total ion chromatograms of BDT in positive ion mode. (C) Baseline chromatogram of the blank sample in positive ion mode. (D-E) Total ion chromatograms of BDT in negative ion mode. (F) Baseline chromatogram of the blank sample in negative ion mode.


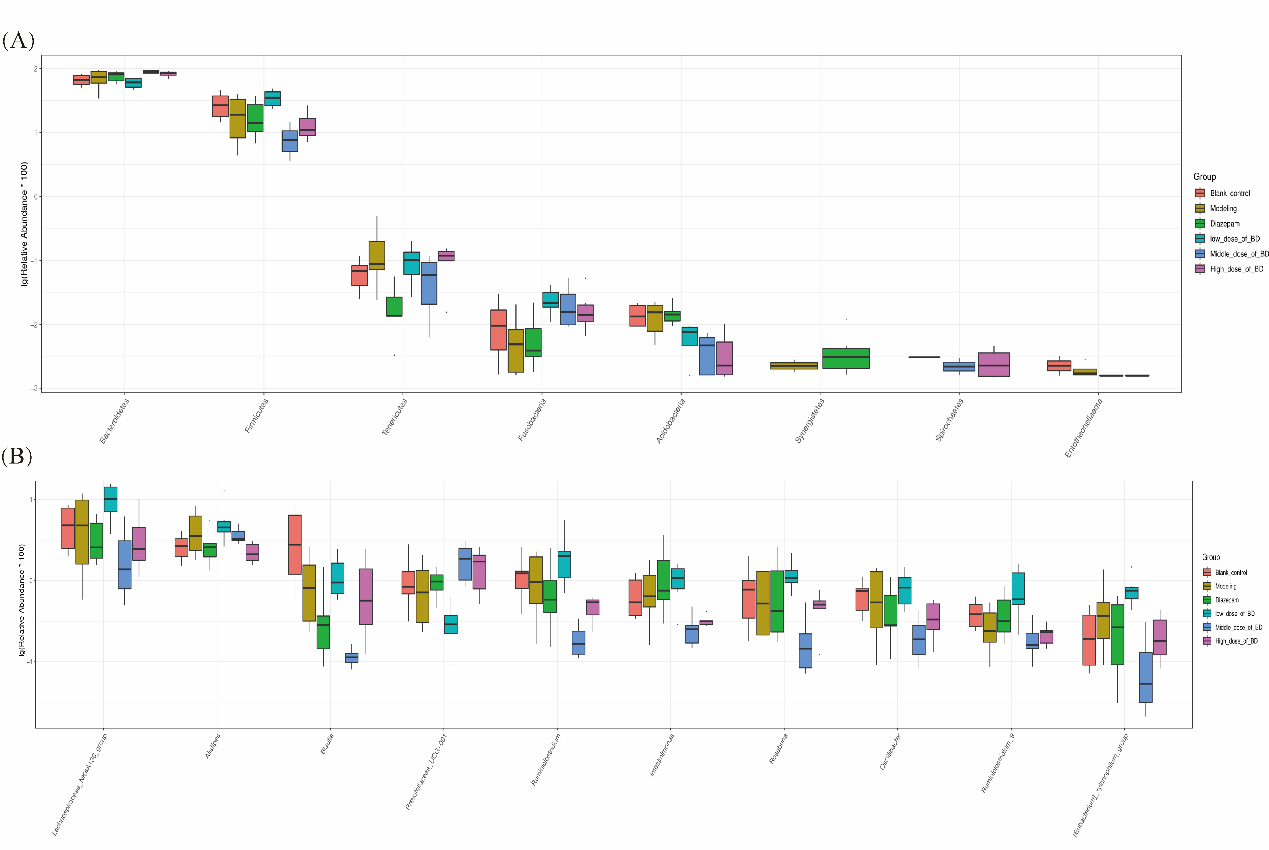


**Figure. S2.** **Comparison of the differences in the relative abundance of gut microbiota between groups at the different levels.** (A) Differences in the top 10 microbial taxa at the phylum level among the groups. (B) Differences in the top 10 microbial taxa at the genus level among the groups.


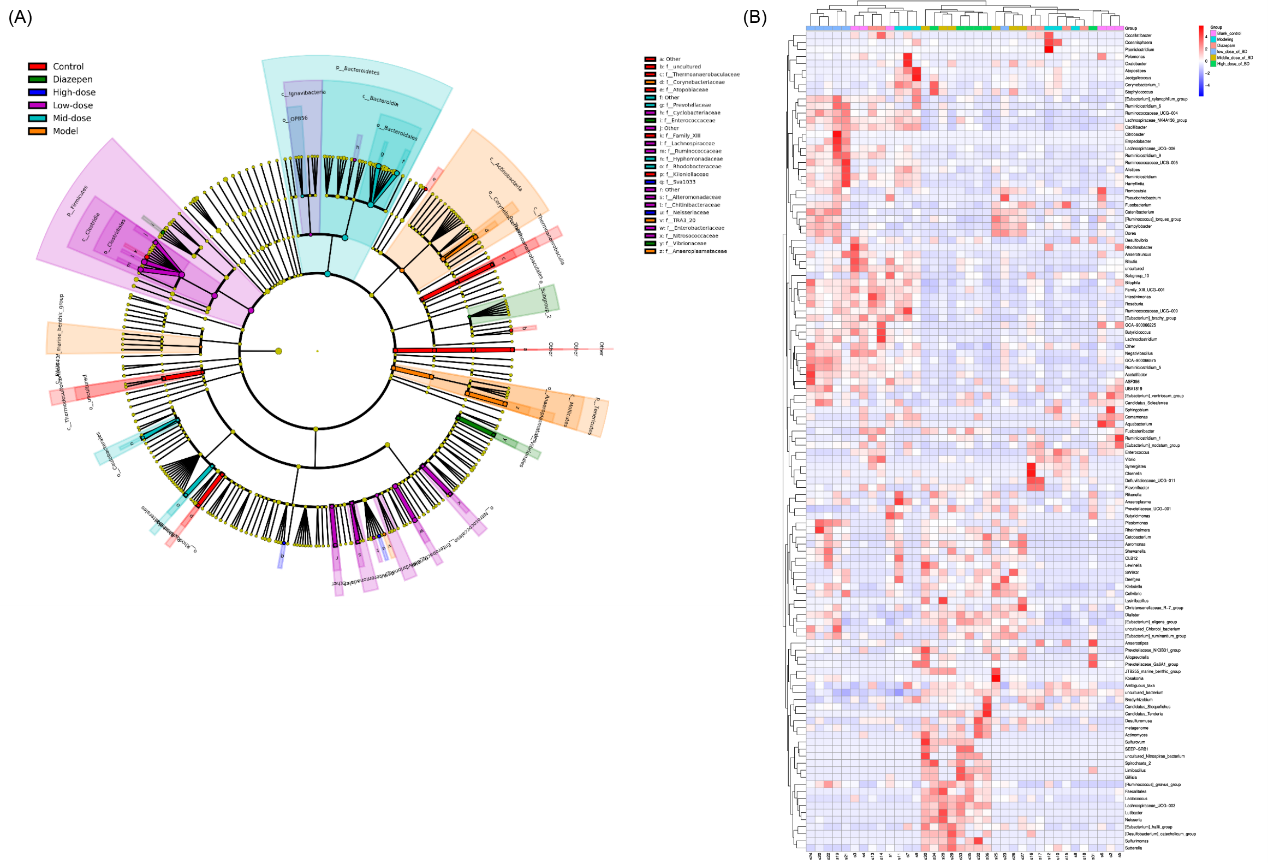


**Figure. S3. Phylogenetic distribution and abundance differences of microbiota across six groups.** (A) Cladogram indicating the phylogenetic distribution of microbiota correlated with the six groups. (B) The differences in abundance among the six groups.
